# Both Low Blood Glucose and Insufficient Treatment Confer Risk of Neurodevelopmental Impairment in Congenital Hyperinsulinism: A Multinational Cohort Study

**DOI:** 10.3389/fendo.2017.00156

**Published:** 2017-07-10

**Authors:** Annett Helleskov, Maria Melikyan, Evgenia Globa, Inna Shcherderkina, Fani Poertner, Anna-Maria Larsen, Karen Filipsen, Klaus Brusgaard, Charlotte Dahl Christiansen, Lars Kjaersgaard Hansen, Henrik T. Christesen

**Affiliations:** ^1^Hans Christian Andersen Children’s Hospital, Odense, Denmark; ^2^Pediatric, Endocrinology Scientific Centre, Russian Academy of Science, Moscow, Russia; ^3^Pediatric Endocrinology, Ukrainian Centre of Endocrine Surgery, Kyiv, Ukraine; ^4^Pediatric, Endocrinology Scientific Centre, Moscow, Russia; ^5^Clinical Genetic Department, Odense University Hospital, Odense, Denmark

**Keywords:** congenital hyperinsulinism, hypoglycemia, neurodevelopmental impairment, neurological outcome, treatment delay

## Abstract

**Background/aims:**

Congenital hyperinsulinism (CHI) is a heterogeneous disease most frequently caused by KATP-channel (ABCC8 and KCNJ11) mutations, with neonatal or later onset, variable severity, and with focal or diffuse pancreatic involvement as the two major histological types. CHI confers a high risk of neurological impairment; however, sparsely studied in larger patient series. We assessed the neurodevelopmental outcome in children with CHI at follow-up in a mixed international cohort.

**Methods:**

In two hyperinsulinism expert centers, 75 CHI patients were included (Russian, *n* = 33, referred non-Scandinavian, treated in Denmark *n* = 27, Scandinavian, *n* = 15). Hospital files were reviewed. At follow-up, neurodevelopmental impairment and neurodevelopmental, cognitive and motor function scores were assessed.

**Results:**

Median (range) age at follow-up was 3.7 years (3.3 months–18.2 years). Neurodevelopmental impairment was seen in 35 (47%). Impairment was associated with abnormal brain magnetic resonance imaging (MRI); odds ratio (OR) (95% CI) 15.0 (3.0–74.3), *p* = 0.001; lowest recorded blood glucose ≤1 mmol/L; OR 3.8 (1.3–11.3), *p* = 0.015, being non-Scandinavian patient, OR 3.8 (1.2–11.9), *p* = 0.023; and treatment delay from first symptom to expert center >5 days; OR 4.0 (1.0–16.6), trend *p* = 0.05. In multivariate analysis (*n* = 31) for early predictors with exclusion of brain MRI, treatment delay from first symptom to expert center >5 days conferred a significantly increased risk of neurodevelopment impairment, adjusted OR (aOR) 15.6 (1.6–146.7), *p* = 0.016, while lowest blood glucose ≤1 mmol/L had a trend toward increased risk, aOR 3.5 (1.1–14.3), *p* = 0.058. No associations for early vs. late disease onset, K_ATP_-channel mutations, disease severity, focal vs. diffuse disease, or age at follow-up were seen in uni- or multivariate analysis.

**Conclusion:**

Not only very low blood glucose, but also insufficient treatment as expressed by delay until expert center hospitalization, increased the risk of neurodevelopmental impairment. This novel finding calls for improvements in spread of knowledge about CHI among health-care personnel and rapid contact with an expert CHI center on suspicion of CHI.

## Introduction

Congenital hyperinsulinism (CHI) is a heterogeneous disease characterized by inappropriate hypersecretion of insulin from pancreatic islet β-cells resulting in hypoketotic hypoglycemia (1, 2). The incidence is estimated to 1:40,000–50,000 (3–5). Although rare, CHI is the most common form of persistent hypoglycemia of infancy and the most difficult to manage (6).

Mutations in at least ten genes have been identified to cause CHI (*ABCC8, KCNJ11, GLUD1, GCK, HADH1, UCP2, HNF4A, HNF1A, SLC16A1, PHOX2B*), most commonly in *ABCC8* or *KCNJ11* expressing the two subunits of the pancreatic β-cell K_ATP_-channel, the sulfonylurea receptor 1, and the potassium inward rectifier 6.2 (Kir6.2) (7–11).

Focal CHI occurs in approximately 40% and is defined as a restricted pancreatic area with adenomatous β-cell hyperplasia, resulting in a lesion composed of confluent islet of Langerhans (9, 12). Focal CHI is strongly associated with a heterozygous, paternally inherited K_ATP_-channel germline mutation combined with a focal loss of heterozygosity on chromosome 11p15 with loss of maternally expressed tumor suppressors, *ABCC8 and KCNJ11* (2). 18F-DOPA PET/CT scan is the preferred method for preoperative localization of the focal lesion (13).

Diffuse CHI involves β-cells throughout the pancreas and is histologically characterized by hypertrophy of a few β-cells per islet. Recessive inactivating mutations in *ABCC8* and *KCNJ11* are the most common causes of medically unresponsive diffuse CHI, whereas dominantly inherited *ABCC8/KCNJ11* mutations typically are medical responsive (2, 14). First line medical treatment after iv glucose and/or glucagon rescue is the K_ATP_ agonist diazoxide with the somatostatin analog octreotide as second line (15). Rapamycin, an mTOR inhibitor, has more recently been used with variable effect (16, 17).

The primary treatment goals are to increase and maintain blood glucose concentration above 3.5, or 3.9, mmol/L, depending on the reference (10). Although heterogeneous in phenotype, CHI often presents in the first days of life with severe and persistent hypoglycemia, representing a severe threat especially for brain metabolism. Furthermore, the elevated serum insulin level results in suppression of ketones bodies, which usually serve as alternative fuel to the brain in the absence of glucose (2, 18, 19) As glucose is required for the rapid growth of the brain, neonates and infants with CHI are especially susceptible to hypoglycemic damage (20, 21). Hypoglycemic convulsions and loss of consciousness are frequent and may be recurrent if not treated sufficiently. Abnormal neurodevelopment outcome has previously been reported in children with CHI, especially motor and speech delay in early childhood (22–30), but also epilepsy, visual impairment, and infantile spasms have been reported (31). Abnormal neurodevelopmental outcome has also been described for children with transient hyperinsulinism, comparable to persistent hyperinsulinism (32, 33).

It could be hoped that the increased recognition in the literature of CHI as a high-risk condition for brain damage has led to a higher prevalence of intact neurodevelopmental outcome today. However, systematic cohort follow-up studies of the neurodevelopmental outcome are sparse (27, 32, 34), especially studies that have clinically testing and neuro-pediatric assessments.

We, therefore, aimed to evaluate the neurodevelopmental outcomes for CHI patients from Scandinavia, Russia, and Eastern Europe including formal testing of cognitive development and motor function when possible.

## Materials and Methods

An international cohort of children diagnosed with CHI was established in collaboration between the Endocrinology Scientific Centre, Russian Academy of Science, Moscow, Russia, and Hans Christian Andersen Children’s Hospital, Odense University Hospital, Denmark, the latter institution also treating patients from other Scandinavian countries, Eastern Europe, and Russia.

The cohort consisted of Scandinavian children, diagnosed, treated, and assessed at follow-up in Denmark at Hans Christian Andersen Children’s Hospital, Odense University Hospital; children from Russia who were diagnosed, treated, and assessed at follow-up in Russia, Endocrinology Scientific Centre, Russian Academy of Science, Moscow; and non-Scandinavians referred from Eastern Europe countries or Russia for further treatment in Denmark. The patients were treated according to international recommendations but with local variations in expertise (expert referral centers/local hospitals), drug availability, and preference. 18F-Fluoro-DOPA PET/CT was only performed in Odense, Denmark, in the period studied. Patients were recruited in the 4-year period January 2013 to December 2016.

The inclusion criteria were persistent (>1 month) CHI with inappropriately elevated p-insulin concentrations during hypoglycemia, defined as blood glucose below 2.5 mmol/L (age ≤3 days), or below 3.2 mmol/L (age >3 days). Exclusion criteria were prematurity [gestational age (GA) <32 weeks] and severe asphyxia.

From the hospital files, the following data were extracted: GA (weeks + days), birth weight, umbilical cord blood pH, and base excess, Apgar score at 1 and 5 min, age at first recorded hypoglycemia episode, blood glucose values and simultaneous beta cell peptides, maximal glucose infusion rate, age at CHI diagnosis, age at treatment start at a local hospital and at a CHI expert center, medical and surgical treatment, genetic investigations (rapid K_ATP_-channel analysis; if negative followed by analysis of other CHI-related genes or next generation sequencing panel for nine known CHI genes), and 18F-Fluoro-DOPA PET/CT results.

Note was made of the treatment delay (in days) from first hypoglycemic sign to first hospital admission, and from first symptom to a CHI expert center. Neurological data were extracted from the hospital files including hypoglycemic seizures, electroencephalography (EEG) reports, diagnoses of epilepsy, head circumference (defining microcephaly <−2SD), blindness, hearing loss, cerebral palsy, neuropsychological development by clinical investigation, and magnetic resonance imaging (MRI) of the brain. In Odense University Hospital, all neurological investigations since 2011, except hearing and eye examination, were performed per protocol and not by indication. The latest reported data were used when appropriate.

After data collection, the children were categorized into severe or moderate disease. Severe CHI was *a priori* defined with one of the following criteria; maximal intravenous glucose >10 mL/kg/min; one or more episodes of hypoglycemia ≤1.0 mmol/L; or homozygous, compound heterozygous, or paternal heterozygous K_ATP_-channel pathogenic mutations with no indication of dominant disease. If none of the above parameters were satisfied, the patients were categorized non-severe CHI.

Congenital hyperinsulinism was categorized as early onset when the first suspected hypoglycemic symptoms occurred <30 days of age; late onset >30 days.

Our cerebral clinical outcomes at follow-up included neurological examination by an expert pediatric neurologist, Bayley Scales of Infant and Toddler Development 3rd Edition (Bayley-III), Developmental Profile 3 (DP-3), Movement Assessment Battery for Children 2 (Movement ABC-2), and Wechsler’s Intelligence Scale for Children fourth edition (WISC-IV) when possible and appropriate. In addition to the follow-up investigations of CHI patients, we included Danish healthy siblings as controls when possible.

Pediatric neurologists, two physiotherapists, and a child psychologist performed the assessments. Bayley-III was used to assess fine and gross motor function, language (receptive and expressive), cognitive, and adaptive development at ages 1–42 months. The Russian children tested by a Russian pediatric neurologist tested all Bayley items. In Denmark, a child physiotherapist tested motor function only. Raw scores of successfully completed items were converted to scale scores and to composite scores (mean of 100) and transformed to a percentile score with a mean of 50.

Developmental Profile 3 was used for assessment of children in Russia from 2 to 6 years of age. DP-3 includes 180 items grouped into scores on adaptive behavior, social–emotional behavior, cognitive function, and communication. Scaled scores were transformed to mean standard scores. A DP-3 standard score of 100 is average and <70 denotes delayed development. A pediatric neurologist interviewed the parents to obtain this assessment.

Motor function was evaluated using Movement ABC-2. This test is suitable for examining 4–12 year-old children’s skills in three core areas: fine motor function, gross motor skills, and static/dynamic balance. The test provides a raw score and standard score, which is transformed to a percentile score with a mean of 50.

Cognitive function was evaluated in Danish children using WISC-IV, of which all 10 core subtests were administered. The full-scale IQ score and 4 index scores were derived (verbal comprehension, perceptual organization, processing speed, and working memory). These scales have population means of 100 and SDs of 15. WISC-IV is appropriate from the age of 6–16 years, 11 months. A single-blinded pediatric psychologist with experience in examining children conducted the WISC-IV testing and scoring.

After testing, the patients were categorized as with normal neurological development, e.g., Bayley-III or Movement ABC-2 ≥5 percentile, DP-3 score ≥70, or WISC-IV developmental quotient ≥70; or with neurodevelopmental impairment, e.g., Bayley-III or Movement ABC-2 score <5 percentile, DP-3 score <70, WISC-IV score <70, blindness, cerebral palsy, or epilepsy. Children who were not able to complete the testing due to severe neurological sequela, such as major intellectual disability or major motor impairment, were categorized to the neurodevelopmental impairment group.

### Statistical Methods

Means were compared using an independent-sample *t*-test or Mann–Whitney, Mantel–Haenszel, or Fisher’s Exact test were appropriate. A *p-*value <0.05 was considered as statistical significant and a trend was considered for *p*-values 0.05–0.10. The association between potential predictive values and mild or severe neurodevelopmental impairment was analyzed. Covariates included lowest blood glucose value, treatment delay from first symptoms to admission at a local hospital or to an expert CHI center (analyzed as categorical values after search for optimal cut-offs), focal vs. diffuse, and CHI disease severity. Univariable regression analysis was followed by multivariable, backward logistic regression models to obtain adjusted odds ratios (aORs) for associations. Data were analyzed using Stata (StataCorp, College Station, TX, USA).

### Ethics

The study was conducted according to the Helsinki Declaration. The Regional Committees on Health Research Ethics for Southern Denmark approved the study, protocol number S-20120119 and by the local Ethical Committee at the Endocrine Research Center of the Ministry of Health Care in Russian Federation.

Written informed consent was obtained from the parents/legal guardians of all research participants. The data were approved by the Danish Data Protection Agency (13/11991).

## Results

The study included 75 children with CHI, Scandinavian group; *n* = 15, referred non-Scandinavians; *n* = 27, Russian group; *n* = 33. In non-Scandinavians, GA and disease severity were higher, and age at follow-up was lower, compared to Scandinavians, Table 1. In total, mean (SD) GA was 38 (2.3) weeks; birth weight 3,553 (730) g. The median (range) onset of first symptoms was ** (**-**) days. Early onset CHI (<1 months) was seen in 50 (67%; median ** days) and late onset in 25 (33%; median ** days). The mean (SD) lowest recorded blood glucose for the total cohort was 0.9 (0.7) mmol/L. Genetic analysis was performed in *n* = 67 patients and K_ATP_-channel mutations were identified in 58%, most frequently in non-Scandinavians, trend *p* = 0.054. No genetic variations were found in 32% of investigated patients. Medical treatment at last hospital contact included diazoxide (*n* = 44), octreotide (*n* = 16), long-acting release octreotide (*n* = 2), and mTOR inhibitor (*n* = 1). Forty (54%) children underwent an 18F-DOPA PET/CT scan, of which 15 (38%) had a focal CHI diagnosis by scan. All 15 patients with a focal CHI by 18F-DOPA PET/CT had restricted focal surgery with histology-confirmed focal lesion and were normoglycemic after surgery.

**Table 1:** Patient characteristics.

	Total	Scandinavian^a^	Referred non-Scandinavian^b^	Russian^c^	*p*-value^e^
Number of patients	*n* = 75	*n* = 15	*n* = 27	*n* = 33	
Birth weight gram, mean ± SD	3,553 ± 730	3,244 ± 586	3,634 ± 787	3530 ± 715	0.13
Gestational age, week, mean ± SD	38 ± 2.3	36 ± 1.0	39 ± 2.3	38 ± 2.1	0.02
Lowest blood glucose, mmol/L, mean ± SD	1.1 ± 0.55	1.2 ± 0.9	0.89 ± 0.6	1.1 ± 0.5	0.17
Glucose infusion rate, mg/kg/min, mean ± SD	10.8 ± 6.6	10.8 ± 6.2	11.0 ± 6.8	N/A	0.75
Early onset <30 days, *n* (%)	50 (67%)	10 (67%)	22 (81%)	18 (55%)	0.18
Late onset >30 days, *n* (%)	25 (35%)	5 (33%)	5 (19%)	15 (45%)	
Age at follow up, median (range), days	750 (46; 6,660)	2,160 (529; 5,400)	278 (46; 6,660)	765 (120; 2,940)	0.00
Severity of disease					0.00
Severe disease^d^, *n* (%)	53 (72%)	8 (53%)	23 (85%)	23 (70%)	
Non-severe disease, *n* (%)	22 (28%)	7 (47%)	4 (15%)	10 (30)%	
Genetic mutations, number of patients	*n* = 67	*n* = 12	*n* = 27	*n* = 28	0.054^f^
*ABCC8, n* (%)	32 (48%)	5 (42%)	16 (59%)	11 (39)%	
*KCNJ11, n* (%)	7 (10%)	0 (0%)	4 (15%)	3 (11%)	
*GCK, n* (%)	0 (0%)	0 (0%)	0 (0%)	0 (0%)	
*GLUD1, n* (%)	5 (8%)	1 (8%)	1 (4%)	3 (11%)	
*HNF1A, n* (%)	0 (0%)	0 (0%)	0 (0%)	0 (0%)	
*HNF4A, n* (%)	1 (1%)	1 (8%)	0 (0%)	0 (0%)	
11p15 uniparental disomy (BWS), *n* (%)	1 (1%)	1 (8%)	0 (0%)	0 (0%)	
No mutations detected	21 (32%)	4 (34%)	6 (22%)	11 (39%)	
Medication at follow-up					
Diazoxide	*n* = 44	*n* = 4	*n* = 9	*n* = 31	
mg/kg/day, mean ± SD	10.8 ± 8.7	23.4 ± 4.4	11.3 ± 4.5	9.2 ± 6.5	
Octreotide	*n* = 16	*n* = 1	*n* = 11	*n* = 4	
mcg/kg/day, mean ± SD	11.8 ± 5	14.6	11.0 ± 5.7	13.5 ± 3.2	
Sandostatin LAR, number of patients	*n* = 2	*n* = 1	*n* = 1	*n* = 0	
Sirolimus, number of patients	*n* = 1	*n* = 0	*n* = 0	*n* = 1	
Treatment delay, from first symptom to	*n* = 55	*n* = 7	*n* = 23	*n* = 25	
Public hospital, median in days; range	3 (0; 780)	0 (0)	0 (0; 180)	30 (0; 780)	0.18
Expert center, median in days; range	11 (0; 2,519)	0 (0; 341)	33 (0; 572)	240 (28; 2,519)	0.10
18F DOPA PET/CT scan, *n*	*n* = 40	*n* = 10	*n* = 25	*n* = 5	
Focal, *n* (%)	15 (38%)	2 (20%)	13 (52%)	0 (0%)	
Diffuse, *n* (%)	25 (62%)	8 (80%)	12 (48%)	5 (100%)	
Surgery, *n*	*n* = 75	*n* = 15	*n* = 27	*n* = 33	
Restricted focal surgery, *n* (%)	15 (19%)	2 (13%)	13 (48%)	0 (0%)	
Pancreatic resection, partial or subtotal, *n* (%)	10 (14%)	5 (33%)	3 (11%)	2 (6%)	
No surgery, *n* (%)	50 (67%)	8 (53%)	11 (41%)	31 (94%)	

*^a^Children born, treated and tested in Scandinavia (One child is Danish citizen but was born in Singapore)*.

*^b^Children from Eastern Europe/Russia treated in Denmark (Belarus, Kazakhstan, Syria and Ukraine)*.

*^c^Children from Russia tested and treated in Russia*.

*^d^Severe disease: hypoglycemia ≤1.0 mmol/L; glucose infusion rate >10 mL/kg/min, or K_ATP_-channel mutations with compound heterozygosity, homozygosity, non-dominant paternal heterozygosity. BWS, Beckwith–Wiedemann syndrome*.

*^e^Scandinavian vs. non-Scandinavian*.

*^f^K_ATP_-channel defect vs. others*.

By clinical examination, six patients (8%) had microcephaly. Abnormal EEG was seen in 13 out of 55 (24%). MRI of the brain was performed in 40, of which in 19 (47.5%) had abnormal findings. White matter changes (hypoglycemic encephalopathy) was seen in 11 (27.5%) and cerebral atrophy in nine (22.5%) as the most frequent findings, Table 2. Infarctions were seen in two patients, probably after prolonged convulsions.

**Table 2:** Brain magnetic resonance imaging (MRI) findings in children with congenital hyperinsulinism.

	Number	Percentage
Total	40	
Normal MRI	21	52.5
Abnormal MRI^a^	19	47.5
Cerebral atrophy		
Generalized	9	22.5
Localized	0	
Infarction		5
Unilateral	2	5
Bilateral	0	
Intracerebral hemorrhage	0	
White matter changes	11	27.5
Occipital	1	2.5
Parieto-occipital	5	12.5
Periventricular	1	2.5
Generalized	4	10

*^a^Four patients had more than one abnormality*.

### Follow-up Investigations

The median age for the study group at the time of follow up was 3.7 years (range 3.3 months to 18.2 years). At the clinical follow-up, 10 patients (14%) had cerebral palsy, Table 3. Epilepsy was seen in 15 (20%), of which one was diagnosed with infantile spasms. Four (5%) had severe visual impairment; none had diagnosed hearing loss. Twenty-three (31%) had severe developmental delay based on clinical neurological examination. In total, 35 (41%) were categorized with severe neurodevelopmental impairment based on the above parameters.

**Table 3:** Clinical neurological outcome for children with congenital hyperinsulinism.

	Total	Scandinavian^a^	Referred non-Scandinavian^b^	Russian^c^
Cerebral palsy, *n* = 75	10 (13%)	1 (7%)	6 (18%)	3 (11%)
Visual impairment, *n* = 75	4 (5%)	0 (0%)	1 (4%)	3 (11%)
Epilepsy, *n* = 75	17 (23%)	2 (13%)	6 (18%)	9 (33%)
Microcephaly, *n* = 39	6 (15%)	1 (7%)	5 (15%)	N/A
Electroencephalography, *n* = 55				
Normal	42 (76%)	3 (75%)	16 (48%)	23 (85%)
Abnormal	13 (24%)	1 (25%)	6 (18%)	6 (22%)
MRI of the brain, *n* = 40				
Normal	21 (53%)	5 (33%)	11 (33%)	5 (19%)
General atrophy	9 (23%)	1 (7%)	7 (21%)	1 (4%)
White matter changes^d^	11 (28%)	1 (7%)	8 (24%)	2 (7%)

*^a^Children born, treated and tested in Scandinavia*.

*^b^Children from Eastern Europe/Russia, tested and further treated in Denmark*.

*^c^Children from Russia, tested and treated in Russia*.

*^d^Reduced myelinization or hypoglycemic encephalopathy*.

### Bayley Scales of Infant and Toddler Development

In the Bayley-III test, mean (SD) total motor function in 43 infants equaled the 29.5 (30) percentile, Table 4. The majority (*n* = 23, 58%) had a normal motor function ≥5 percentile with a mean (SD) of 53.4 (21.3) percentile. Fifteen (35%) were categorized as impaired by total motor score with a mean (SD) of 0.9 (1.4) percentile.

**Table 4:** Bayley-III, Developmental Profile 3 and Movement ABC-2 test results for children with congenital hyperinsulinism.

	Russian^a^	Referred non-Scandinavian^b^	Scandinavian^c^
Bayley-III, mean ± SD, percentiles	*n* = 13	*n* = 27	*n* = 3
Cognitive	20.3 ± 31.6		
Communication	13.1 ± 20.3		
Motor	29.0 ± 31.5	27.1 ± 31.0	50.0 ± 0
Social-emotion	23.4 ± 32.9		
Adaptive	15.6 ± 33.9		
Developmental Profile 3, mean ± SD, score	*n* = 20		
Physical	75.6 ± 26.5		
Adaptive	77.6 ± 28.1		
Social	75.3 ± 28.6		
Cognitive	70.5 ± 26.0		
Communication	73.5 ± 25.6		
Total score	68.2 ± 31.4		
Movement ABC-2, mean ± SD, percentiles			*n* = 12
Fine motor			32 ± 37
Gross motor			52 ± 39
Balance			38 ± 29
Total			37 ± 35

*^a^Children from Russia, tested and treated in Russia*.

*^b^Children born in Eastern Europe/Russia, tested and further treated in Denmark*.

*^c^Children born, treated, and tested in Scandinavia*.

Mean (SD) cognitive Bayley-III score was 20 (31.6) in 13 infants, of which four had a mean <5 p denoting neurodevelopmental impairment. For communication, social–emotional behavior and adaptive behavior, mean (SD) percentile varied from 13 (20.3) to 23.4 (32.9). Based on all available Bayley-III data, 18 (42) were categorized as with neurodevelopmental impairment.

### Developmental Profile 3

In the DP-3 test performed in Moscow in 20 children, mean (SD) full-scale score was 68 (31), which denoted to below average. Eight children (40%) were categorized as normally developed with a mean (SD) of 100 (23.8). Twelve children (60%) had a DP-3 score below 70 denoting neurodevelopmental impairment. Of these, nine had a score <50 denoting severe neurodevelopmental delay. The sub-items physical, adaptive, communication, cognitive, and social profile had a mean (SD) score from 77 (28) to 70 (26).

### Movement ABC-2

Age-standardized motor function percentiles in the Danish patients aged >6 years could be compared to sibling controls (*n* = 5), thereby reducing the impact of parental factors. A significantly lower mean total motor function was observed in the CHI group, 38 ± 35 vs. 78 ± 35 percentile, *p* = 0.047, Table 5. This was driven by a significant lower balance function for the CHI patients, 38 ± 29 vs. 72 ± 26 percentile (*p* = 0.03), and to a less degree a reduced fine motor function, 32 ± 37 vs. 65 ± 34 percentile (trend *p* = 0.10). No significant difference was seen in gross motor function. Two children had a total motor function <5 p that denoted to the neurodevelopmental impairment group and two children had low normal total motor function score equaling 5–15 p.

**Table 5:** Cognitive score and motor function for children with congenital hyperinsulinism above 6 years of age and healthy siblings as controls.

	Patient 1/sibling	Patient 2 (sibling)	Patient 3 (sibling)	Patient 4	Patient 5	Patient 6	Patient 7 (sibling)	Patient 8	Patient 9	Patient 10	Patient 11	Patient 13
**WISC-IV score**												
Verbal comprehension index	99/110	97/99	122/118	91	99							
Perceptual organization index	79/120	101/105	122/111	96	109							
Working memory index	60/134	83/77	83/86	92	116							
Processing speed index	92/130	110/144	101/121	87	84							
**Total score**	**79/126**	**96/106**	**112/112**	**89**	**101**							
**Movement ABC, percentile**												
Fine motor	5/95	84/75	1/63	5	84	37/84	5/9	8	5	63	25	99
Gross motor	2/84	75/95	9/75	16	95	84/95	5/25	91	5	98	84	75
Balance	37/91	91/91	91/91	9	50	37/50	16/37	63	37	50	16	25
**Total**	**5/98**	**91/95**	**9/84**	**5**	**91**	**50/95**	**1/16**	**63**	**10**	**63**	**25**	**75**

*WISC-IV: Wechsler’s Intelligence Scale for Children fourth edition*.

### WISC-IV

Cognition was assessed by use of WISC-IV for five patients, of which three had a healthy sibling tested for control, Table 5. The mean (SD) full-scale score for the five patients were 95.6 (12.4) with a range of 79–112.

For the three tested sibling-pairs, a trend toward a nine score lower mean was observed for the CHI patients, 95 ± 12 vs. 114 ± 10 (*p* = 0.066). This trend was driven by a difference in the processing speed, 80 ± 14 vs. 131 ± 7, *p* = 0.054. All CHI children were on basis of this assessment denoted to the normally developed group.

### Overall Outcome

The overall neurodevelopmental outcome is listed in Table 6. Of our total cohort, 47% (*n* = 35) had neurodevelopmental impairment. Low normal function by Bayley-III or movement ABC-2 equaling 5–15 percentile was detected in six (8%).

**Table 6:** Overall neurodevelopmental outcome for children with congenital hyperinsulinism (CHI) by patient data.

	Normal development	Neurodevelopmental impairment
Geography and center category	40 (53%)	35 (47%)
Scandinavians	12 (80%)	3 (20%)
Eastern Europeans	12 (44%)	15 (56%)
Russians	16 (36%)	17 (52%)
Onset		
Early onset	24 (48%)	26 (54%)
Late onset	16 (64%)	9 (36%)
Medical delay from first symptom		
≤2 days	19 (61%)	12 (39%)
>2 days	21 (48%)	23 (52%)
Medical expert delay from first symptom		
≤5 days	12 (80%)	3 (20%)
>5 days	28 (47%)	32 (53%)
Lowest blood glucose		
Blood glucose ≤1 mmol/L	10 (33%)	20 (67%)
Blood glucose >1 mmol/L	30 (67%)	15 (33%)
Severity of disease		
Non-severe CHI	15 (68%)	7 (32%)
Severe CHI	25 (47%)	28 (53%)
Genetics		
K_ATP_-channel mutations	17 (44%)	22 (56%)
Other gene mutations	6 (86%)	1 (14%)
No mutations detected	11 (52%)	10 (48%)
18F-DOPA PET/CT scan		
Focal	6 (40%)	9 (60%)
Diffuse	14 (56%)	11 (44%)
N/A	21 (60%)	14 (40%)
Magnetic resonance imaging brain		
Normal	16 (76%)	5 (24%)
Abnormal	3 (16%)	16 (84%)
N/A	21 (60%)	14 (40%)
Age at follow-up		
Age ≤810 days	18 (50%)	18 (50%)
Age >810 days	22 (56%)	17 (44%)

*Normal development: WISC-IV score >70, Bayley-III or Movement ABC-2 >15 percentile, or DP-3 score >70. Neurodevelopmental impairment: WISC-IV score <50, Bayley-III or movement ABC-2 <5 percentile, Developmental Profile 3 score <50, epilepsy, cerebral palsy, visual impairment, or too retarded to complete testing*.

Split by nations, 20% of the Scandinavian patients had neurodevelopmental impairment, whereas 52% of the Russian patients, and 56% of the non-Scandinavian patients referred to Denmark were severely impaired. All six children with microcephaly were denoted to the neurodevelopmental impairment group. All of those belonging to the normally developed group had a normal EEG, whereas 45% of those with neurodevelopmental impairment had an abnormal EEG. Three children with occipital hypoglycemic encephalopathy by MRI scan showed normal development at follow-up.

In univariable analysis, neurodevelopmental impairment was associated with abnormal MRI of the brain; OR (95% CI) 15.0 (3.0–74.3), *p* = 0.001, non-Scandinavian patient, OR 3.8 (1.2–11.9), *p* = 0.023; treatment delay from first symptom to expert center >5 days; OR 4.0 (1.0–16.6), trend *p* = 0.05, and lowest blood glucose ≤1 mmol/L; OR 3.8 (1.3–11.3), *p* = 0.015, Tables 6 and 7. Patients with K_ATP_-channel mutations and focal disease had marginal trends only toward higher odds of impairment. No associations were detected for early vs. late disease onset, disease severity, delay from first symptom to first treatment, or age at follow-up.

**Table 7:** Logistic regression for factors associated to neurodevelopmental impairment in congenital hyperinsulinism (CHI) patients.

	Univariate regression		Multivariate regression			
Factors	Crude OR (95% CI)	*p*-value	a(OR) Model 1 *n* = 31	*p*-value	a(OR) Model 2 *n* = 31	*p*-value
Non-Scandinavian vs. Scandinavian, *n* = 75	3.8 (1.2–11.9)	**0.023**				
Early vs. late onset, *n* = 75^a^	1.0 (0.4–2.8)	0.97				
Medical delay >2 vs. ≤2 days, *n* = 56	1.5 (0.5–4.4)	0.42				
Expert delay >5 vs. ≤5 days, *n* = 56	4.0 (1.0–16.6)	**0.050**	4.4 (0.7–28.4)	0.12	15.6 (1.6–146.7)	**0.016**
Lowest blood glucose ≤1 vs. >1 mmol/L, *n* = 63	3.8 (1.3–11.3)	**0.015**			3.5 (0.9–12.7)	**0.058**
Severe vs. non-severe CHI, *n* = 74^b^	1.6 (0.6–4.1)	0.35				
K_ATP_-channel mutations vs. not, *n* = 68	1.6 (0.2–8.5)	0.11				
Focal vs. diffuse disease, *n* = 39	2.8 (0.7–11.1)	0.13				
Magnetic resonance imaging brain normal vs. abnormal, *n* = 38	15.0 (3.0–74.3)	**0.001**	16.8 (2.3–123.3)	**0.005**		
Age at follow up, >810 vs. <810 days, *n* = 74	2.1 (0.62–7.5)	0.22				

*Model 1 included all factors before stepwise exclusion. Model 2 did not include MRI of the brain and age at follow-up*.

*OR, odds ratio; a(OR), adjusted odds ratio*.

*^a^Early onset ≤30 days; late onset >30 days*.

*^b^Severe CHI: maximal intravenous glucose >10 mL/kg/min, one or more episodes of glucose ≤1 mmol/L, or homozygous, compound heterozygote or paternal, non-dominant heterozygous K_ATP_-channel mutations*. *Significant or trend p-values in bold*.

In the multivariable analysis, only 31 patients could be included due to missing data. In Model 1, only MRI of the brain remained associated to neurodevelopmental impairment, aOR (95% CI) 16.8 (2.3–123.3), *p* = 0.005, while “expert delay” had a trend toward increased risk, OR 4.4 (0.7–28.4), *p* = 0.12. When analyzing associations to early predictors only by exclusion of age at follow-up and MRI of the brain (Model 2), delay from first symptom to expert center treatment significantly associated with neurological impairment, aOR 15.6 (1.6–146.7), *p* = 0.016, whereas lowest blood glucose ≤1 mmol/L conferred a trend toward increased risk, aOR 3.5 (0.9–12.7), *p* = 0.058.

## Discussion

In our mixed international cohort of children with CHI, 47% of the patients had neurodevelopmental impairment at follow-up, despite huge advances in knowledge about CHI. Univariable factors associated with neurodevelopmental impairment were delayed from first symptom to expert treatment center (trend), lowest recorded blood glucose ≤1.0 mmol/L, non-Scandinavian patient, and abnormal MRI of the brain. By multivariable regression analysis, excluding brain MRI, “expert delay” became significantly associated, while lowest recorded blood glucose ≤1.0 mmol/L showed a trend toward increased risk. Compared to siblings, motor function and cognitive score were lower in a small subset of Danish patients.

Neonatal hypoglycemia is a well-known cause of neurodevelopmental impairment, depending on the hypoglycemia severity, duration, and comorbid factors (35–38). Neonatal hypoglycemia is, however, usually transient and predictable by perinatal risk factors, which confers opportunities for prevention and early management (39). Early detection and adequate treatment of transient neonatal hypoglycemia are associated with an un-increased risk of adverse neurological outcome (37), or lower fine motor function in boys and a higher internalizing score in girls, both within the normal range at school age follow-up. Other follow-up studies report of increased adjusted odds for developmental delay in late preterms with blood glucose <1.1 mmol/L (40), adverse neurodevelopmental outcome in diabetic mothers’ term neonates with neonatal blood glucose 0.0–1.5 mmol/L at 7–8 years’ follow-up (41), and lower school performance at 10 years after early transient hypoglycemia (<2.5 mmol/L < 3 h of age) in preterm and term neonates (42).

In CHI, an alarmingly high prevalence of neurodevelopmental delay, epilepsy, seizures, blindness, and microcephaly have been reported in other studies (22–24, 31, 34, 43–46). Of note, even children with transient CHI had a 30% risk of abnormal neurodevelopment in a recent UK study (32), with onset <7 days of age and disease severity defined by diazoxide dose as predictive factors. Other studies of various size and outcomes have shown a risk of adverse neurological outcome in CHI up to 50% (22, 26–28, 30, 34, 46–48).

Of larger follow-up studies, neurodevelopmental retardation was found in 44% of 114 CHI patients in Germany (23), psychomotor retardation in 26% of 90 patients in France (22), neurological deficits in 45% of 65 CHI patients in Australia (48), and neurodevelopmental delay in 18% of 43 Saudi Arabian CHI patients (47). Despite the huge improvements in knowledge and management possibilities in CHI, our multinational patient cohort showed no improvement in neurodevelopmental outcome compared to the former studies.

We also found that motor function, especially balance and fine motor function, and cognitive score were lower, although still in normal range, in a small subset of Danish patients compared to their siblings. Similar findings were seen in follow-up of Danish children with transient neonatal hypoglycemia (unpublished data). This indicate that even apparently normal functioning children with CHI should be fine-tested at follow-up, as some could benefit from interventions to improve especially balance and fine motor function.

In our study, abnormal MRI of the brain strongly associated to neurodevelopmental impairment, also in the multivariable analysis. Hypoglycemia of any kind in the neonatal period or infancy is associated to abnormal MRI findings, especially white matter changes also known as hypoglycemic encephalopathy (49–51). In CHI, posterior (parieto-occipital) white matter changes is especially prominent, which relate to timing of hypoglycemia to the neonatal period and lack of ketone bodies as alternative fuel (51) We also found a high prevalence of posterior white matter lesions, but in the most severe, undertreated patients, global cerebral atrophy, and intracerebral hemorrhage were seen.

In the Saudi study of 43 patients, only 14 out of 18 patients with developmental delay had cerebral atrophy on imaging (47), stating that not all with abnormal cerebral imaging will present with clinically overt impairment, as also seen in our study.

Among early predictors, recorded blood glucose ≤1.0 mmol/L significantly associated with neurodevelopmental impairment. In contrast to others (22, 23, 32), we detected no increased cerebral risk for early onset CHI, neither when using a cut-off of 30 days, nor 7 days (data not shown). We believe that untreated hypoketotic hypoglycemia is the major direct cause of brain damage in CHI. Although the neonatal brain is more susceptible to hypoglycemia than in later childhood, early onset may well be a proxy for severe hypoglycemia, which is strongly associated to early onset, but difficult to extract without information bias in a retrospective setting with data from many hospitals in many countries, where frequent and systematic blood glucose recordings may not have been the rule. Our *a priori* defined severe CHI category did not associate with the neurological outcome. We never the less succeeded in detecting an association to adverse outcome for lowest recorded blood glucose ≤1.0 mmol/L. The definition of severe CHI included an i.v. glucose demand >10 mg/kg/min, which we used as an alternative to lowest blood glucose <1.0 mmol/L, as the latter criteria was subjected to variations in treatment. However, initial i.v. glucose demand recordings were often missing and hence recorded if possible at follow up before 18F-DOPA PET/CT (performed without other medication).

In our mixed cohort, delay until expert center and non-Scandinavian patient status were also determining factors for neurodevelopmental impairment in univariable analysis. This was probably due to non-recorded low and/or long hypoglycemic periods in this group of patients. As hypoglycemia should be treated promptly and sufficiently, these factors may, therefore, reflect insufficient treatment. However, the multivariate analysis only had delay until expert center as a significant associated factor. This may be explained by a lower study number in the multivariate analysis, but also by the fact that non-Scandinavian patients had a higher disease severity than Scandinavians, possibly reflecting selection bias. Considering delay until expert treatment, this may also reflect both selection bias and unrecorded insufficient treatment (low blood glucose). However, selection bias could be bidirectional: Patients with mild CHI may not have been referred to an expert center, but on the other hand, some patients with very severe disease were referred promptly. This leaves insufficient treatment as the major likely explanatory factor. The range of documented insufficient treatment was in one non-Scandinavian patient 12 months with untreated, very severe CHI, and cerebral palsy and severe infantile spasms. The insufficient treatment in this patient reflected limited hospital resources and self-payment for medical treatment.

In the other end of the spectrum, a Danish term neonate was erroneously discharged untreated a few hours after birth despite an unmeasurable low blood glucose recording because of paucity of symptoms, but readmitted at the age of 2 days with loss of consciousness and convulsions and blood glucose of 0.0 mmol/L. Only 1 week later, this patient was referred to the national expert center. Despite adequate treatment from then on, this infant also showed neurodevelopmental impairment and infantile spasms.

The novel identification of insufficient treatment as a risk factor does not call for more knowledge about CHI, but for improvements in spread of knowledge about CHI among health care personnel, especially rapid contact with an expert CHI center on suspicion of CHI.

Already in year 2000, a workshop held by the European network for research into hyperinsulinism (ENRHI) advocated for early referral to a multidisciplinary expert center (15). National or international referral is nowadays frequent for this rare and complex disease with need of experts in 18F-DOPA PET, medical, and surgical management. However, our findings underscore the need of significant improvements in prompt diagnosis and adequate treatment of CHI in every birth clinic and hospital, as just a very short time without sufficient treatment can cause brain damage. Given the rarity and often surprising severity of CHI, this task remains a major challenge for health-care providers dealing with birth clinics, neonatal, and pediatric wards, for health-care authorities and for relevant medical societies. Education of health-care personnel and popular communication should be systematically enforced, potentially in the context of hypoglycemia in general. Force task groups should be set to develop strategies for the prevention of untreated or undertreated CHI. Last, rapid funding is urgently needed in order to avoid delay in international referrals when needed.

Limitations of our study included the observational, partially retrospective design, selection bias due to referral practice favoring more severely affected patients and those with genetic results suggesting focal CHI, and the cohort size, however, relatively large for a rare disease. Lack of systematic data included not only brain MRI but also initial blood glucose values, continuous glucose monitoring, and i.v. glucose demand recordings. The disease severity assessment was, therefore, subjected to limitations. Follow-up was performed at different ages by different investigators using different methods, why robust cut offs (normal/abnormal) were used for comparisons. At last follow-up, some of the patients had relatively low age, where neurodevelopmental adverse outcomes may not yet be detectable. Multivariable analyses were hampered by missing data and hence a lower study number, and residual confounding, including potential unmeasured factors related to local differences in our mixed cohort, e.g., socioeconomic factors.

Strengths included our multinational setting, hospital file review of blood glucose data, the detailed and systematical prospective neurological outcome testing at follow-up, and the relatively long median follow-up time.

In conclusion, 47% of our CHI patients had neurodevelopmental impairment at follow-up, despite huge advances in knowledge about CHI. Not only very low blood glucose but also insufficient treatment as expressed by delay until expert center hospitalization increased the risk of neurodevelopmental impairment. This novel finding calls for improvements in spread of knowledge about CHI among health-care personnel and rapid contact with an expert CHI center on suspicion of CHI.

## Ethics Statement

The study was conducted according to the Helsinki declaration. The Regional Ethical Committee approved the study, protocol number S-20120119. The data were approved by the Danish Data Protection Agency (13/11991).

## Author Contributions

AH collected and analyzed the data and wrote the manuscript. MM, EG, IS, FP, CC, KF, A-ML, LH, and KB have collected and interpreted data and written on the manuscript. HC initiated and supervised the study and supervised the manuscript writing.

## Conflict of Interest Statement

The authors declare that the research was conducted in the absence of any commercial or financial relationships that could be construed as a potential conflict of interest.
